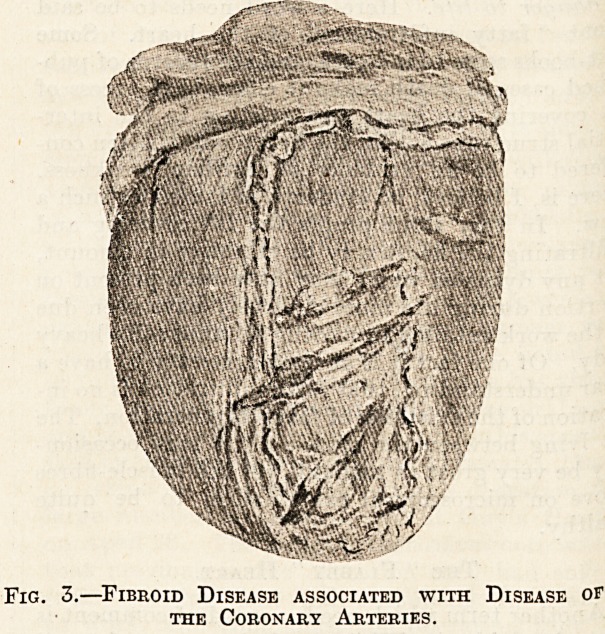# Some Affections of the Heart Concerned in Sudden Death

**Published:** 1907-05-11

**Authors:** Theodore Fisher


### Hospital Clinics.

### SOME AFFECTIONS OF THE HEART CONCERNED IN SUDDEN DEATH.

By THEODORE FISHER, M.D., M.R.C.P.

### III. THE CARDIAC MUSCLE.

In reports of inquests a frequent cause for death is said to be "fatty heart." There is generally much misunderstanding in the minds of those who use the term as to the nature of a fatty heart. A heart in which the main morbid condition present is what has been called fatty degeneration is comparatively rare, and except as a complication of diphtheria may be said virtually never to be a cause of sudden death. In passing, a word as to the nature of fatty degeneration may not be out of place. Modern physiology seems to indicate that fat is not formed from proteids, and that consequently fat does not arise in cardiac muscle fibres as a result of destruction of the important elements of these fibres. Most of the fat which may become evident in certain conditions of disease was present in the heart before the disease arose, but changes occur as the result of deleterious influences which render the fat visible to the naked eye or to histological methods of examination. Should the deleterious influences act only for a short time and be removed before death takes place the heart generally completely recovers. In other words, fatty degeneration when existing alone —apart from any other forms of degeneration of the cardiac muscle—must be looked upon as a temporary abnormal condition; that is to say, *there is no such disease as fatty disease of the heart, which may last for months and years and be a source of danger to life.* Here a word needs to be said about "fatty infiltration" of the heart. Some text-books seem to indicate, and the records of published cases of death make it clear, that excess of fat covering the heart and existing in the interstitial structures within the heart-wall is often considered to be an evidence of cardiac weakness. There is, I believe, no evidence in favour of such a view. In very stout people the fat covering and infiltrating the heart may be excessive in amount, but any dyspnœa which may have been present on exertion during life must obviously have been due to the work entailed in moving an unusually heavy body. Of one fact it is at least necessary to have a clear understanding, that fatty infiltration is no indication of the existence of fatty degeneration. The fat lying between the muscle-fibres may occasionally be very great in amount, yet the muscle-fibres prove on microscopical examination to be quite healthy.

### The "Flabby" Heart.

Another term which needs a word of comment is that of "flabby." While the designation "fatty" indicates a pathological condition of the cardiac muscle that under certain conditions may exist, the term "flabby" has no pathological meaning. The heart may be found after death to be firm or flabby, but those who consider a soft condition of the heart to be an evidence of disease overlook the consequences of changes which occur in the body after death. The cardiac muscle, like the voluntary muscles, undergoes rigor mortis. During rigor mortis the muscle-wall of the ventricles contracts. The wall of the strong left ventricle in contracting generally expels most of the blood it contained at the time of death, but the weaker right ventricle more often fails to drive out so much of the blood within it. Consequently there may not infrequently be seen a firmly contracted and conical left ventricle, and a right ventricle more or less distended. Should rigor mortis have passed off instead of the firm heart with a conical left ventricle, there will be a soft heart, which, after removal from the body,

Fig. 1.—"Tabby-cat" Striation of a Muscular Pillar.

flattens out when laid on the post-mortem table. *A "flabby" heart, therefore, is a heart from which rigor mortis has disappeared.* The early onset of flabbiness will depend not on the condition of the cardiac muscle, but upon the heat of the weather and the nature of the illness which has led to death. In enteric fever, for example, the heart is far more likely to be found to be flabby at the time of the autopsy than in cerebral hæmorrhage, not because there is fatty degeneration in the case of fever—any such degeneration, if present, will be trifling in amount—but because rigor mortis has already passed away.

To return to fatty degeneration. It is scarcely necessary to refer to the well-known appearance known as "tabby-cat striation" (see Fig. 1), which is most commonly seen in death following anæmia due to severe loss of blood, especially when a septicæmia is associated with the anæmia, such as may occur in puerperal fever following post-partum hæmorrhage. Tabby-cat striation is also not uncommonly seen in cases of rheumatic pericarditis when the pericarditis has existed for two or three

Fig. 2.—Disease of the Aorta Obstructing the Orifices of the Coronary Arteries.

Rough sketch of a portion of the heart of a sailor, aged 46, who died suddenly. Below the aortic valve a cut has been made, which exposes the interior of the inter-muscular septum of the ventricles, and shows that much of the muscle has been replaced by fibrous tissue.

weeks or longer, but diseases such as rheumatism which occasion poisoning of the cardiac muscle more frequently give rise to a diffuse fatty degeneration. Pale, ill-defined patches are then sometimes seen in the heart-wall, the nature of which does not become clear until examination by histological methods has been undertaken, though in many cases nothing abnormal can be detected with the naked eye. Fatty degeneration of this character may be seen in diphtheria, yet in diphtheria where there has been sudden death the amount of fat present in the cardiac muscle is not always very noteworthy, possibly because death has occurred before sufficient time has elapsed for the pathological changes to become evident. In some cases of sudden death in diphtheria death is, however, hastened by paralysis of the diaphragm, and in others probably, as Bolton thinks, poisoning of nerve centres in the floor of the fourth ventricle may play a part in the fatal issue.

It may be repeated that fatty degeneration of the heart, when existing as the most important pathological condition present, occurs as a complication of an acute or subacute disease, either outside or within the heart. Fatty degeneration may, however, be associated with a chronic affection of the heart-wall. This affection is one in which sudden death may occur during apparently perfect health. At the autopsy, fatty changes, however, if present, are generally slight compared with the amount of fibrous tissue. *Fibroid disease of the heart, not fatty degeneration, has long been recognised as being very commonly found in cases of sudden death.* Some fatty changes are not infrequently also present, but the fat is only a stepping-stone towards the replacing of the muscle-fibres by fibrous tissue. Fibroid degeneration of the cardiac muscle is often consequent upon defective nutrition of the heart-wall due to interference with the circulation through the coronary arteries. It was pointed out, when speaking of sudden death in aortic valvular disease, that the most serious cases are those in which there is disease of the first part of the aorta causing obstruction of the orifices of the coronary arteries. It was also mentioned that sudden death may occur when the aorta is diseased, while the cusps of the aortic valve remain healthy and competent. The association of disease of the first part of the aorta with fibroid disease of the cardiac muscle is perhaps the most common pathological condition found in cases of sudden death when death has overtaken an apparently healthy adult engaged in his daily occupation. This is especially the case when the adult is still in middle life.

### Atheromatous Disease.

In later life another cause of fibroid disease of the cardiac muscle becomes more frequent; that is atheromatous disease, not of the orifices of the coronary arteries, but of the vessels themselves. Atheromatous disease of the coronary arteries is, however, common where the cardiac wall remains healthy, and occasionally fibroid disease may be present when there is no obvious obstruction in the arteries. Possibly in the cases where disease of the coronary arteries is absent

Fig. 3.—Fibroid Disease associated with Disease of the Coronary Arteries.

the nutrition of the heart suffers as a consequence of old age rather than from definite local interference with nutrition. Another, but not common, cause of fibrosis of the cardiac muscle should be mentioned; this is general thickening of the arteries throughout the body while, however, the nodular patches known as atheroma are absent. When this general thickening of arteries is present I have seen the cardiac muscle thickly studded with small circular patches of fibrosis resembling grey tubercles. Such an appearance in my experience is not common, but Professor Delepine exhibited a good example in the museum of specimens at the annual meeting of the British Medical Association when held in Manchester.

In cases of fibroid disease of the heart associated with disease of the aorta, or with atheroma of the coronary arteries, the amount of fibrosis of the cardiac muscle varies greatly. In some cases the fibrosis is so extensive that it is difficult to understand how the heart can have continued to act efficiently up till the time of sudden death; while in others the scattered fibroid patches are so small and so few in number that, allowing that they represent the extent of the disease, there seems nothing to explain the abrupt arrest of cardiac action. The fibroid patches may occur as small spots or narrow streaks numerous and widely distributed, or may exist as one or more large patches measuring one inch, or even nearly two inches, across. Fig. 2 represents a portion of the aorta and of the adjacent septum interventriculorum from a sailor, aged 46, who died suddenly. The disease of the aorta which is represented had greatly obstructed the orifices of the coronary arteries. The cut surface of the muscular septum shows grey and dark streaks. The dark streaks are the muscle-fibres; the pale areas the fibrous tissue. Such well-marked fibrosis as this is, however, exceptional. Fig. 3 illustrates fibroid disease of the heart associated with atheroma of the coronary arteries. The terminal branch of the right coronary artery is seen passing over the posterior surface of the left ventricle, and in its neighbourhood incisions into the heart-wall have exposed fibroid patches.

*(To be continued.)*

## Figures and Tables

**Fig. 1. f1:**
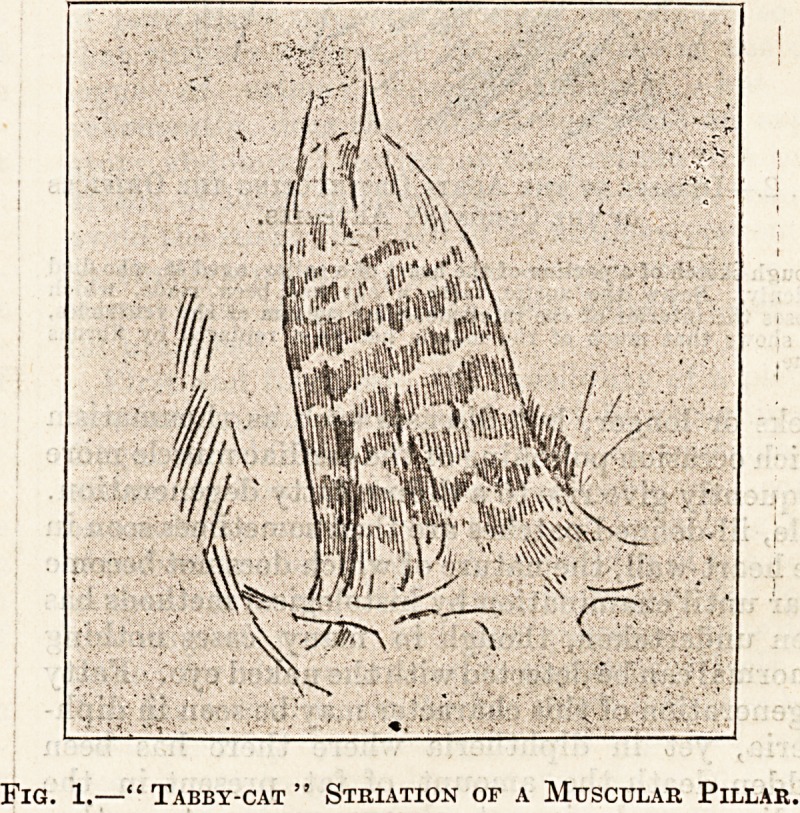


**Fig. 2. f2:**
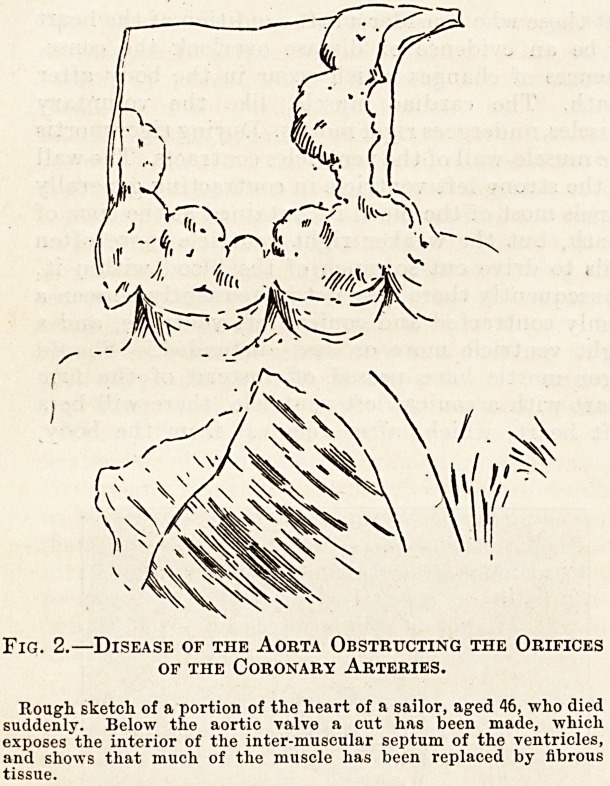


**Fig. 3. f3:**